# Primed T Cell Responses to Chemokines Are Regulated by the Immunoglobulin-Like Molecule CD31

**DOI:** 10.1371/journal.pone.0039433

**Published:** 2012-06-19

**Authors:** Madhav Kishore, Liang Ma, Georgina Cornish, Sussan Nourshargh, Federica M. Marelli-Berg

**Affiliations:** 1 William Harvey Research Institute, Barts and The London School of Medicine and Dentistry, Queen Mary University of London, London, United Kingdom; 2 Division of Medicine, Department of Immunology, Imperial College London, London, United Kingdom; University of Nebraska Medical Center, United States of America

## Abstract

CD31, an immunoglobulin-like molecule expressed by leukocytes and endothelial cells, is thought to contribute to the physiological regulation T cell homeostasis due to the presence of two immunotyrosine-based inhibitory motifs in its cytoplasmic tail. Indeed, loss of CD31 expression leads to uncontrolled T cell-mediated inflammation in a variety of experimental models of disease and certain CD31 polymorphisms correlate with increased disease severity in human graft-versus-host disease and atherosclerosis. The molecular mechanisms underlying CD31-mediated regulation of T cell responses have not yet been clarified. We here show that CD31-mediated signals attenuate T cell chemokinesis both *in vitro* and *in vivo*. This effect selectively affects activated/memory T lymphocytes, in which CD31 is clustered on the cell membrane where it segregates to the leading edge. We provide evidence that this molecular segregation, which does not occur in naïve T lymphocytes, might lead to cis-CD31 engagement on the same membrane and subsequent interference with the chemokine-induced PI3K/Akt signalling pathway. We propose that CD31-mediated modulation of memory T cell chemokinesis is a key mechanism by which this molecule contributes to the homeostatic regulation of effector T cell immunity.

## Introduction

CD31, or platelet endothelial cell adhesion molecule-1 (PECAM-1) is a member of the immunoglobulin gene superfamily expressed at high density at the lateral borders of endothelial cells and at a lower density on the surface of hematopoietic cells including T lymphocytes [1].

CD31–deficient mice exhibit a very mild phenotype and have normal numbers of T cells [2]. However, genetic deletion of CD31 leads to exaggerated disease severity in inducible experimental models of T cell-mediated inflammation, including experimental autoimmune encephalomyelitis (EAE) and collagen-induced arthritis (CIA) [3], [4], suggesting that CD31 signals play a functional regulatory role under conditions of immunological stress. The immunoregulatory role of CD31 interaction has recently begun to be appreciated also in human diseases. Loss of CD31 expression by CD4^+^ T cells correlates with increased size of atherosclerotic aortic aneurism size, a condition in which T cell immunity is a well-established pathogenic factor [5]. In addition, single nucleotide polymorphisms of CD31 encoding amino acid substitutions at positions affecting the binding site [6] and the intracellular ITIMs [7] are associated with increased severity of graft-versus-host disease after hematopoietic stem cell transplantation [8], [9], [10], [11], [12] and atherosclerosis [7], [13]. Although T cell-mediated inflammation contributes to the pathogenesis of both these conditions, the molecular mechanisms underlying this link are at present unclear.

The immunoregulatory activity of CD31 has been correlated with the attenuation of T Cell Receptor (TCR) signalling and reduced Zap-70 phosphorylation, mediated by phosphatases recruited by its ITIM motifs [14], [15], which results in the inhibition of T cell expansion and effector function [15]. This effect however cannot fully account for the uncontrolled inflammation observed in diseased CD31−/− mice, as exaggerated CD31−/− T cell expansion is counterbalanced by enhanced activation-induced T cell death [15], [16].

Enhanced T cell extravasation to non-lymphoid target tissue is a key feature of inflammation as observed in murine models of autoimmunity induced in CD31-deficient mice [3], [4]. It has been suggested that loss of junction integrity by vascular endothelium lacking CD31 expression at non-lymphoid sites of inflammation [17] might trigger this effect. However, the lack of other cardinal signs of vascular leakage in CD31-deficient mice led us to hypothesize that CD31 signalling might directly regulate intrinsic T cell motility under inflammatory conditions.

Inflammatory chemokines are key mediators of T cell infiltration of target tissue [18]. We therefore sought to investigate the effect of CD31 deficiency on T cell responses to chemokines both *in vitro* and *in vivo*. We here show that CD31 signals modulate chemokinesis in activated, but not naïve, T lymphocytes. This effect depends on CD31 membrane clustering and segregation on the memory T cell leading edge, cis-CD31 engagement on the same membrane and subsequent interference with the PI3K/Akt signalling pathway.

## Results

### CD31 Regulates Chemokine-induced T Cell Migration *in vitro* and *in vivo*

In contrast to the increased T cell infiltration of inflamed target tissue by activated CD31−/− T cells, cellular composition and architecture of secondary lymphoid tissue appears to be normal in CD31−/− mice [2]. In addition, localization of adoptively transferred wild-type (WT) and CD31−/− naïve T cells to the spleen is comparable [19]. These observations suggest that migration of naïve and memory T lymphocytes is differentially regulated by CD31 signals.

**Figure 1:**
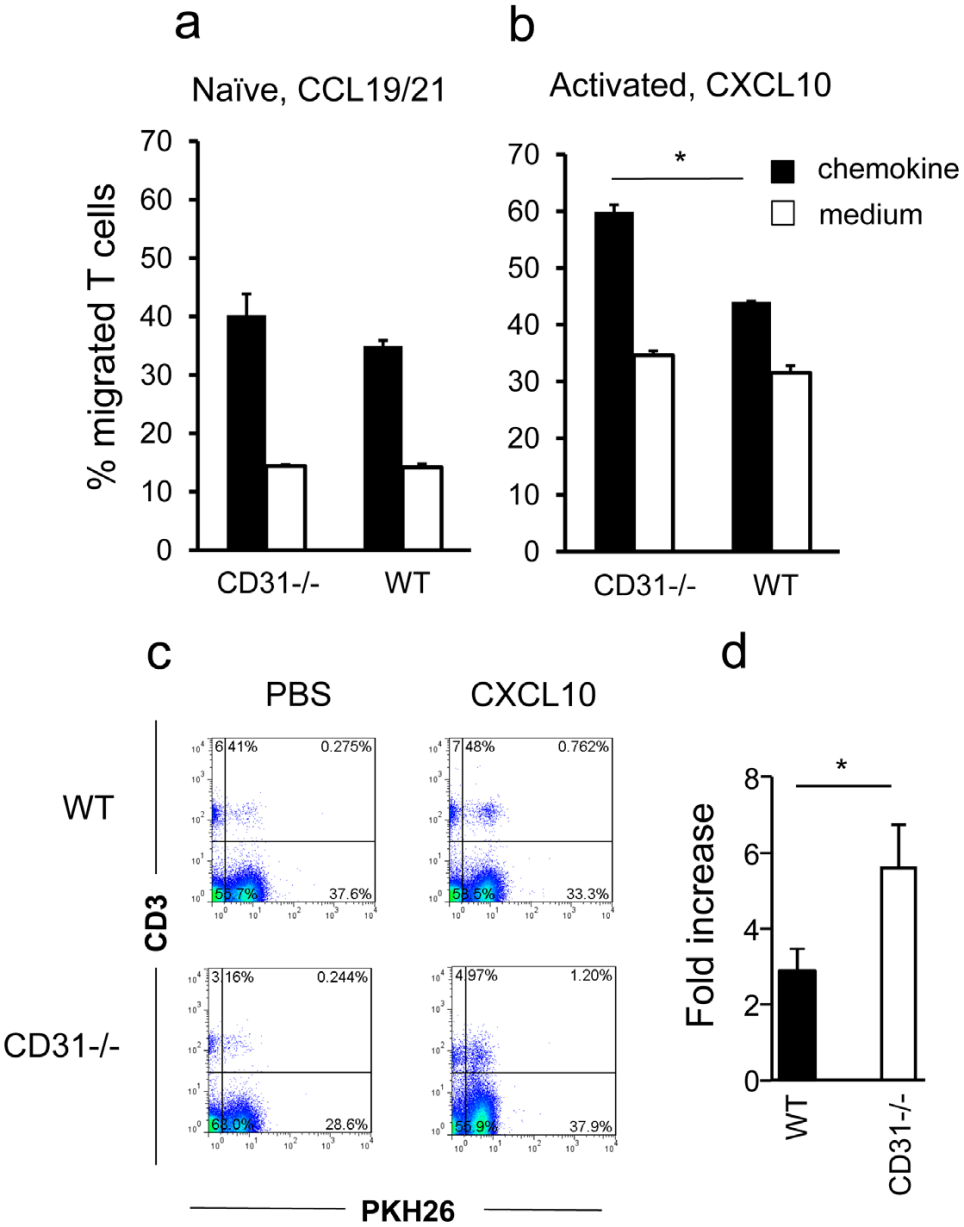
CD31-deficient activated T cells display enhanced responses to chemokines *in vitro* and *in vivo*. Panels a-b: Naïve and activated WT and CD31−/− T cell migration through a transwell in response to the chemokines CCL19/21 and CXCL10, respectively, was assessed over 6 hours. Percentage migration was calculated by dividing the number of cells in the bottom chamber by the original number of cells plated onto the transwell. The average percentage migration from four independent experiments is shown. Error bars indicate SD (*p<0.01). Panel c: Effector memory HY-specific WT or CD31−/− T cells were labeled with PKH26 and injected i.v. into syngeneic female mice that had received an i.p. injection of 1.2 µg CXCL10 30 minutes earlier. Mice were sacrificed 16 hours later, and the presence of PKH26-labeled T cells in the peritoneal lavage was analysed by flow cytometry. Due to the presence of an autofluorescent population of non-T cells often detected in FL-2, cells were double-stained with an APC-conjugated anti-CD3 antibody. Representative dot plots are depicted on the left hand panels. The average fold-increase (T cells in chemokine-treated animals/T cells in PBS-treated animals ± SD) of PKH26 (FL-2)-labeled T cells gated in the CD3^+^ T cell population retrieved from at least five animals/group in 3 independent experiments of similar design are shown on the right hand panels. *p<0.04.

To test this hypothesis, transwell-based assays were set up to compare chemokinesis of WT and CD31-deficient naïve or memory T lymphocytes. Comparison of adhesion and chemokine receptors (including CCR7 and CXCR3) and activation markers expression by naïve and activated T cells did not reveal any difference between WT and CD31−/− T cells (Figure S1). The chemokine ligands for CCR7 and CXCR3 - characteristically expressed by naïve and activated T cells respectively - were used to assess chemokinetic responses by WT and CD31−/− T cells. As it is shown in Figure 1a, CD31−/− naïve T cell migration in response to the chemokines CCL19 and CCL21 was comparable to that by WT T lymphocytes. In contrast, migration of CD31-deficient memory T cells (HY-specific T cell lines) to the chemokine CXCL10 was significantly increased compared to that by their WT counterpart (Figure 1b). This effect was not CXCL10-specific as enhanced chemokinesis by activated T cells to CXCL12 was also observed (Figure S2).

To verify whether this effect was operational *in vivo*, PKH26-labeled WT or CD31−/− HY-specific effector memory T cells were injected intravenously (i.v.) into WT recipients, which had received 1200 ng CXCL10 or saline solution intraperitoneally (i.p.) prior to adoptive transfer, as we have previously described [20]. T cells with a defined antigen specificity were used in these experiments to rule out any interference due to antigen-induced migration [21], [22] – hence HY-specific T cells were injected into female (non-antigenic) syngeneic recipients. Phenotypic characterization of WT and CD31−/− effector memory T cells did not reveal any significant difference in the expression of the array of molecules analyzed (Figure S3). Recruitment of labeled T cells in the peritoneal cavity was assessed 16 hours later by flow cytometric analysis of the peritoneal lavage. As shown in Figure 1c and d, CXCL10-driven localization of CD31−/− T cells was significantly enhanced compared to that by WT T cells, suggesting that loss of CD31 signals leads to increased chemokine-driven extravasation into non-lymphoid tissue. The proportion of WT and CD31−/− CD4^+^ and CD8^+^ T cells in the migrated lymphocyte population was comparable (CD4^+^ T cells: approximately 81±5% WT and 76±6% in CD31−/− T cells; CD8^+^ T cells: approximately 16±3% WT and 18±5 CD31−/− T cells), suggesting that chemotaxis by these T cell subsets is equally affected by CD 31 signalling.

### CD31-mediated Signals Interfere with the Chemokine-induced Akt/PKB Pathway

**Figure 2:**
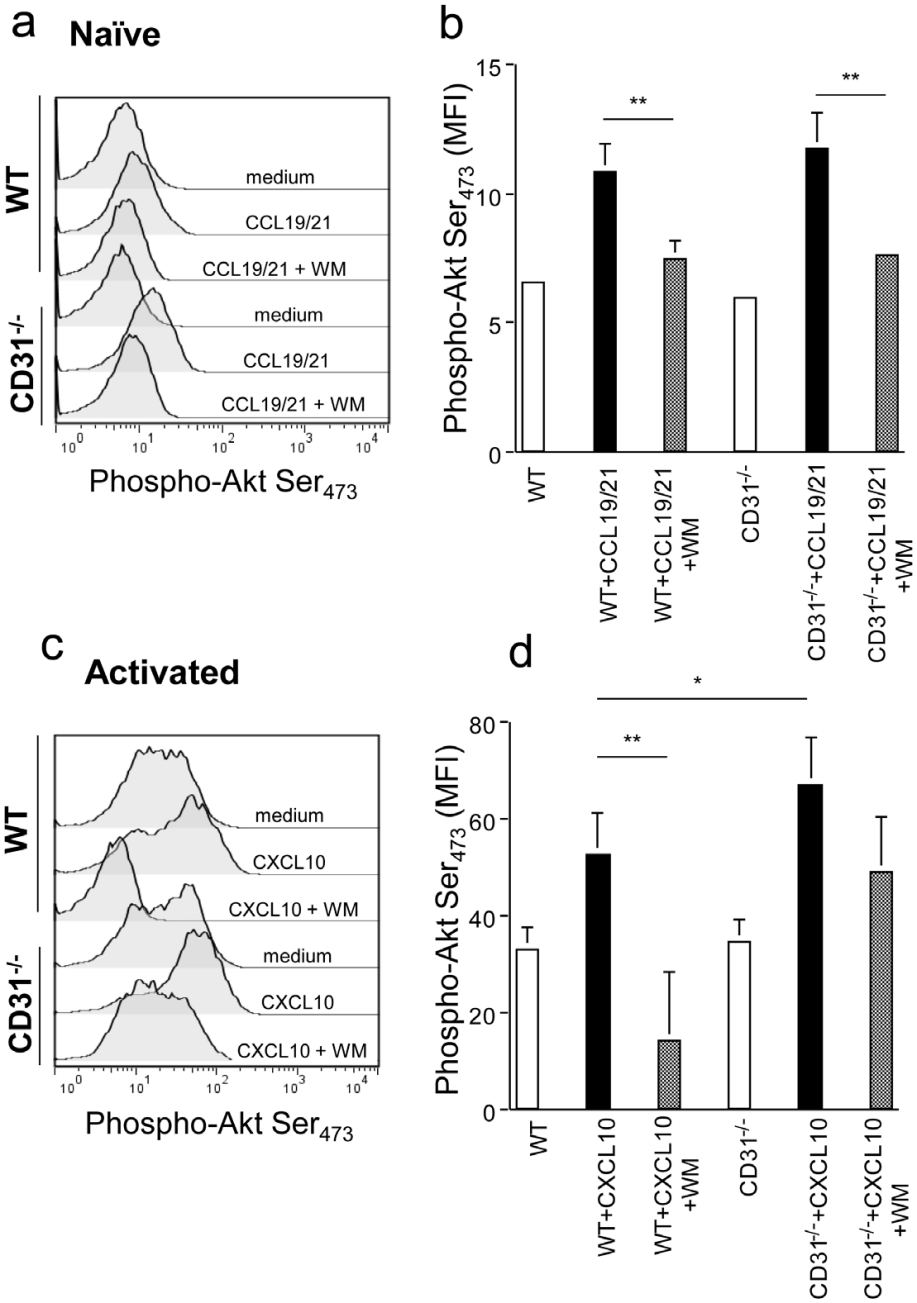
CD31 inhibits chemokine-induced Akt phosphorylation in activated T cells. Naïve and activated T cells were exposed to CCL19/21 and CXCL10, respectively, for 2 minutes. Phosphorylation of Akt at serine 473 was assessed by antibody staining and flow cytometry. In panels a and c. representative histograms of the experimental conditions indicated beside each profile are shown. Panel b and d indicate cumulative data of the mean fluorescence intensity (MFI) indicative of Akt phosphorylation obtained in the various conditions indicated in at least 4 independent experiments of identical design. *p<0.05 **p<0.02.

**Figure 3:**
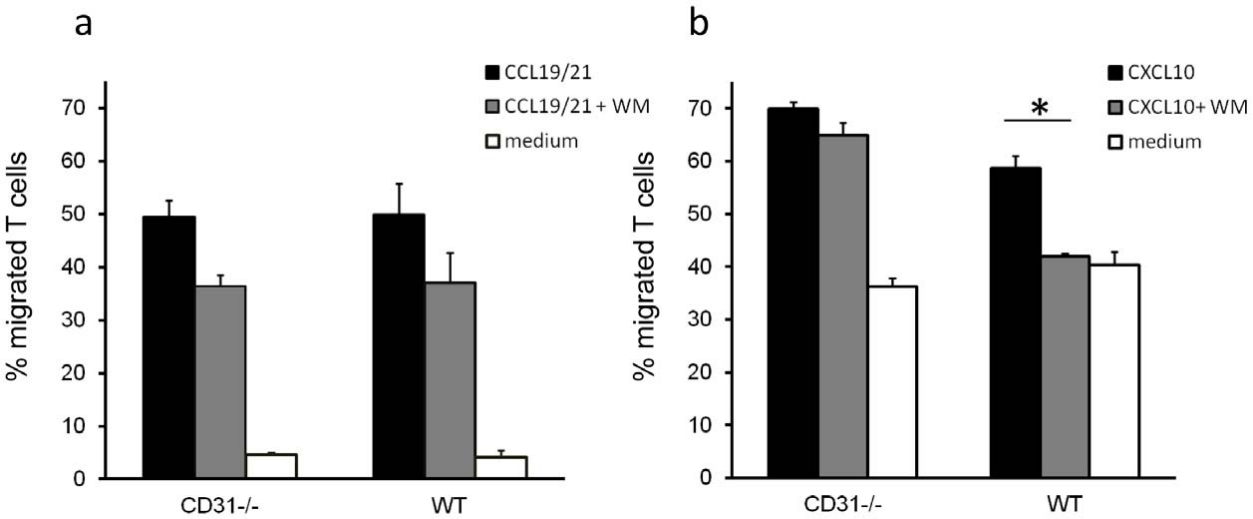
CD31-deficient T cell chemokinesis is partially resistant to PI3K inhibition. Naïve (panel a) and activated (panel b) WT and CD31−/−T cell migration in response to either CCL19/21 (naïve) or CXCL10 (activated) through a transwell was assessed as described in Methods. Some T cells were pre-incubated with the PI3K inhibitor Wortmannin (10 µM) for 30 minutes at RT. Percentage migration was calculated by dividing the number of cells harvested from the bottom chamber following 6 hours incubation at 37°C by the original number of cells plated onto the transwell. The average percentage migration from at least three independent experiments is shown. Error bars indicate SD (*p<0.01).

The main signalling pathway induced by chemokine receptor engagement during chemokinesis involves PI3K-dependent Akt/PKB phosphorylation [18]. As the recruitment of phosphatases is a key feature of CD31 signalling [14], we assessed whether the increased chemotactic activity selectively observed in memory CD31−/− T cells correlated with alterations in Akt phosphorylation. Naïve and antibody-activated T cells (7-day cultures) were exposed to 300 ng/ml CCL19/21 and CXCL10, respectively, for 2 minutes. Akt activation was then measured by staining with an antibody recognizing Akt phosphorylated at serine residue 473. As it is shown in Figure 2a–b, Akt phosphorylation upon chemokine stimulation was comparable in WT and CD31−/− naïve T lymphocytes, and this was prevented by the addition of the PI3K inhibitor Wortmannin. In contrast, Akt phosphorylation was significantly increased in CD31−/− activated T cells exposed to CXCL10 as compared to their WT counterpart (2c–d). The addition of a suboptimal dose of Wortmannin (10 µg/ml) led to inhibition of Akt phosphorylation in WT T cells, while Akt remained largely phosphorylated in CD31−/− T cells. The ‘spread’ pAkt profiles of activated T cells are likely to reflect the heterogeneous signalling responses of primary T cells even following optimal activation, which have been previously reported in a number of studies [23], [24], [25]. These data were further supported by the observation that the same dose of Wortmannin significantly inhibited chemokinesis of activated WT, but not CD31−/−, T cells (Figure 3a–b). Interestingly, despite inhibiting chemokine-induced Akt activation in naïve T cells (Figure 2a–b), exposure to Wortmannin did not significantly diminish CCL19/21-induced naïve T cell chemotaxis (Figure 3a). In this context, naïve T cell homing to secondary lymphoid tissue has been shown to be largely mediated by DOCK2-activation and relatively PI3K-independent [26].

Overall, these data suggest that CD31 signals may attenuate chemokine-induced signals by interfering with Akt phosphorylation in activated T lymphocytes.

### Differential Cellular Segregation of CD31 Molecules in Naïve and Activated T Cells

**Figure 4:**
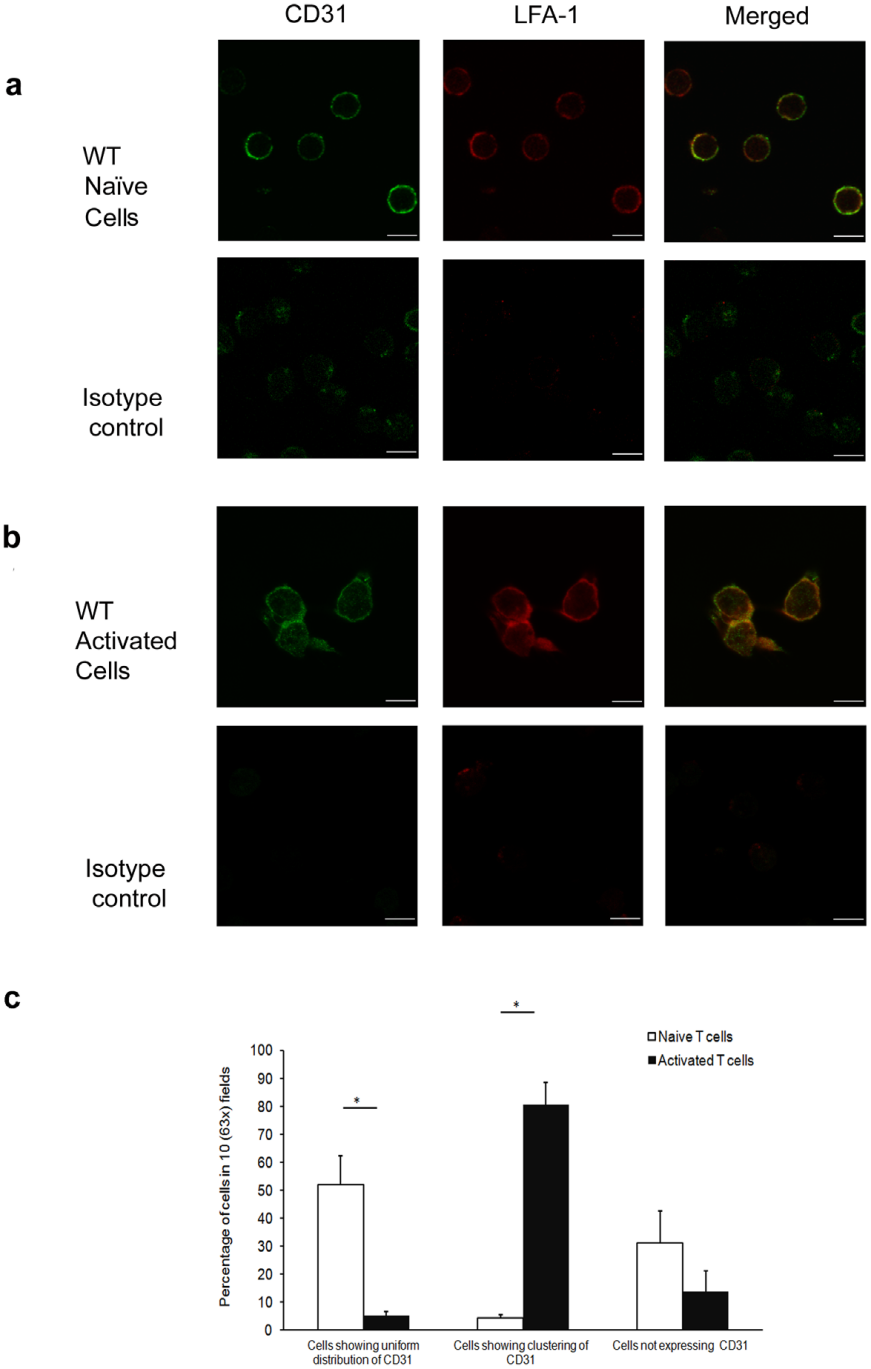
CD31 molecules segregate differently in naïve and activated T lymphocytes. Confocal images of naive WT T cells stained with rabbit-anti-mouse CD31 (green fluorescence) and rat anti- mouse LFA-1 (red fluorescence) followed by incubation with secondary antibodies Alexa Fluor 488-conjugated donkey anti-rabbit IgG and Alexa Fluor 647-conjugated goat anti-rat IgG, respectively, are shown in panel a. Added scale bar = 6 µm. Panel b: WT Activated T cells generated via anti-CD3 and anti-CD28 treatment over 7 days were allowed to rest for 24 hours in low serum and then fixed. LFA-1 and CD31 expression was visualized as described above. Added scale bar = 6 µm. The average CD31 distribution/expression (± SD) from at least four 63× magnified fields obtained in three independent experiments of identical design is shown in panel c (*p<0.001).

The molecular basis of the different effects of CD31-mediated regulation of chemokine-induced signals in naïve and memory T cells was further investigated by analyzing CD31 molecule segregation in these cell types by confocal microscopy. First, naïve and activated T cells were co-stained with an antibody recognizing LFA-1, a surface integrin which is expressed at low levels and homogenously dispersed on the naïve T cell surface, and is upregulated and clustered in activated T cells [27]. As it is shown in Figure 4a, in naïve T cells CD31 was largely localized to the cell membrane, where it was homogenously distributed. In contrast, albeit downregulated, CD31 was predominantly aggregated in large clusters in activated T cells (Figure 4b).

**Figure 5:**
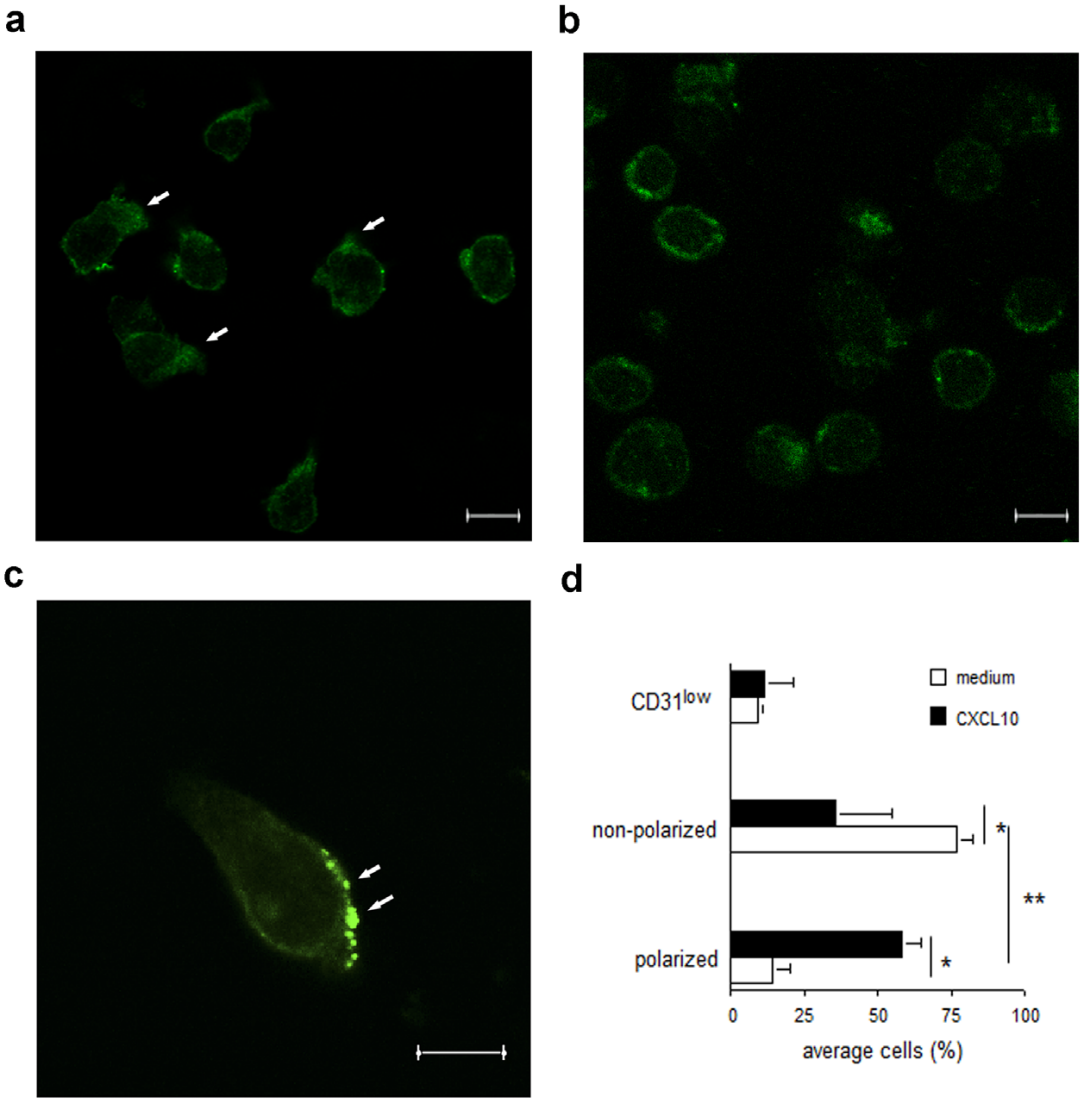
Polarization of CD31 molecules in activated T cells following exposure to chemokines. Activated WT T cells were allowed to migrate through transwells in response to the chemokine CXCL-10 (panel a) or incubated in medium alone (panel b) and then fixed for analysis. Cells were not permeabilized to allow surface staining only. Confocal images of T cells stained with rabbit-anti-mouse CD31 followed by incubation with Alexa Fluor 488-conjugated donkey anti-rabbit IgG are shown in panels a and b. A higher magnification of a migrating lymphocyte further depicting CD31 molecule polarization is shown in panel c. Confocal z stacks series were acquired using a step size of 0.5 µm. Added scale bar = 6 µm. The average CD31 distribution/expression (± SD) from at least four 63× magnified fields obtained in three independent experiments of identical design is shown in panel b (*p<0.001).

Confocal imaging of activated T cells migrating to CXCL10 indicated that CD31 aggregates were largely polarized to the leading edge of migrating T cells (Figure 5 a–b), thus being ideally placed for interference with the biochemical pathways initiated by chemokine receptors, which also segregate to the lamellipodia of migrating T cells [28]. In this context, co-localization of CD31 molecules and phosphatases to the leading edge in migrating granulocytes has been previously reported [29].

### Mechanisms of CD31 Triggering During Chemokinesis

A major issue raised by the observations described above concerns the mechanism of CD31 triggering during chemokinesis. While during trans-endothelial migration homophilic interactions most likely occur between CD31 molecule on EC and T cell apposing membranes, cell:cell contact is unlikely to take place during migration through a transwell. Based on previous report that dynamic CD31 cis-membrane interactions (i.e. clustering within the same cell membrane) can induce signalling events in CD31-transfected human embryonic kidney and erythroleukemia cells [30], we reasoned that CD31 molecule segregation in compact clusters on the memory T cell surface (Figure 4b, d) might elicit similar effects.

**Figure 6:**
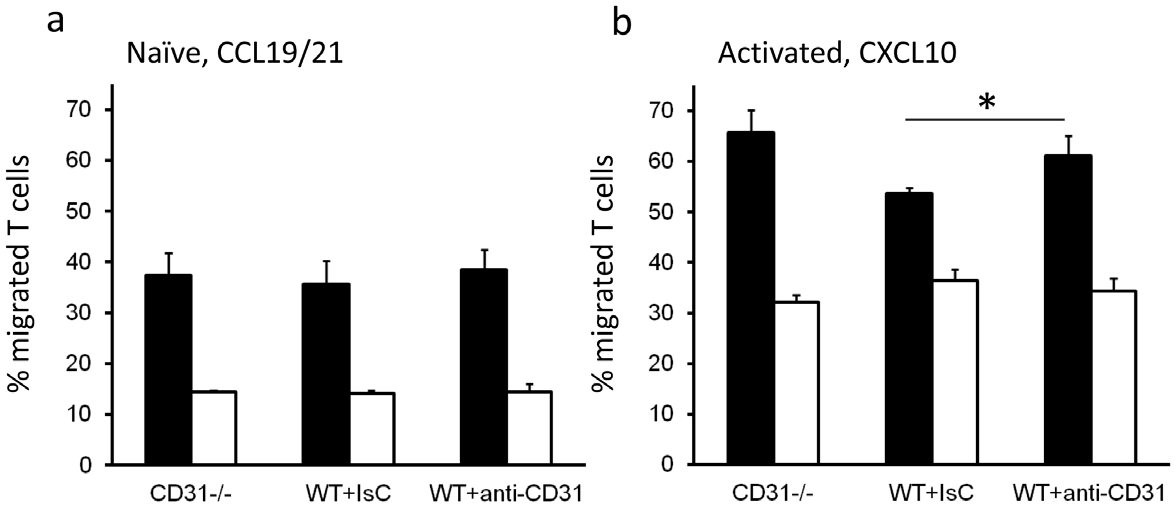
Antibody-mediated CD31 ‘immobilization’ enhances activated T cell chemokinesis. Naïve (a) and activated (b) WT T cells were incubated overnight in RPMI 0.5% FCS. Some T cells were pre-incubated with an anti-CD31 mAb at saturating concentrations (5 µg/ml) for 30 minutes at RT. Migration in response to CXCL10 or medium through a transwell was assessed over 6 hours. Percentage migration was calculated by dividing the number of cells harvested from the bottom chamber following 6 hours incubation at 37°C by the original number of cells plated onto the transwell. The average percentage migration from three independent experiments is shown. Error bars indicate SD (*p<0.05).

We therefore sought to investigate whether interfering with this molecular segregation could enhance T cells response to chemokines in the absence of intercellular CD31 engagement. As the CD31 domain responsible for cis-interactions has not been identified, we decided to disrupt CD31 oligomerization by steric interference. WT naïve and activated T cells were ‘rested’ in serum-free medium overnight and then treated with an anti-CD31 antibody known to inhibit CD31 homophilic interactions without inducing signalling [31], in order to prevent cis-membrane re-clustering. An isotype-matched antibody was used as a control. Naïve and activated T cells were then exposed to CCL19/21 and CXCL10, respectively, in a transwell chemokinesis assay. As it is shown in Figure 6a, antibody pre-treatment did not affect migration of naïve T cells, while anti-CD31-treated activated T cells displayed significantly enhanced chemokinesis (Figure 5b). It is known that hemophilic engagement of CD31 by dendritic cells leads to functional CD31 signalling in naive T cells [15].

Together with the observation that CD31 molecules segregate in compact clusters on activated T cell surface these data are consistent with the hypothesis that CD31 interactions on the same cell membrane are required to inhibit chemokinesis.

## Discussion

In this study, we show that CD31 signals attenuate T cell responses to chemokines, a previously unknown function of this immunoreceptor. Importantly, the regulation of response to chemokines by CD31 selectively affects primed T cells, in which this molecule segregates in membrane clusters.

This spatial distribution favours molecular interactions on the same cell membrane, which we propose as a mechanism inducing CD31 signalling in polarized, migrating T cells. This hypothesis is supported by our observation that steric interference, achieved with a non-activating antibody, prevents modulation of T cell chemokinesis by CD31.

Dynamic CD31 cis-membrane homophilic interactions have been previously described in CD31-transfected human embryonic kidney and erythroleukemia cells [30], although the molecular domain/s mediating these interactions have not been identified. As activated T cells do not express any other known CD31 ligand [32], it is likely that the effect that we observe is due to CD31 interactions with itself, although we cannot formally exclude the possibility that CD31 is associated with a different protein.

The enhanced chemokinetic responses by CD31-deficient primed T cells correlate with increased phosphorylation of mediators of the PI3K/Akt pathway and partial resistance to PI3K inhibition. A key feature of CD31 is its ability to recruit phosphatases following its engagement [14]. It is therefore likely that CD31-dependent modulation of chemokinesis is mediated by the recruitment of phosphatases targeting this pathway. In this context, it has been recently reported that silencing of SHIP-1, a phosphatase recruited by CD31 [33], leads to increased basal phosphorylation of protein kinase B (PKB)/Akt and its substrate GSK3β, as well as an increase in basal levels of polymerized actin following chemokine stimulation of primary activated human T cells [34].

In naïve T cells, despite being expressed at higher levels, CD31 is evenly distributed on the cell surface, and does not interfere with chemokine induced Akt phosphorylation. Similarly, naïve T cell responsiveness to chemokines is unaffected by loss of CD31 signals. In line with this observation, naïve T cell numbers and distribution are unaffected by genetic deletion of CD31 [2], [16].

The physiological relevance of the distinct effects of CD31 signalling on naïve and activated T cell responsiveness to chemokines must lay, in our view, in their different trafficking patterns and proliferative potential. Naïve T cells continuously recirculate through secondary lymphoid tissue in response to constitutively SLO-expressed chemokines such as CCL19 and CCL21 [35]. This type of trafficking does not generate inflammation. However, CD31 signalling becomes functional in naïve T cells when engaged in trans by dendritic cells, with the effect of modulating their excessive expansion during priming [15].

Upon antigen activation, T cells are re-programmed to respond to inflammation-induced chemokines, such as CXCL10, which allow them to gain access to non-lymphoid tissue where they can induce damage [35]. The proliferative potential of memory T cells is much reduced as compared to that of naïve T cells and possibly does not require substantial regulation for homeostatic purposes.

Thus, we propose that CD31 regulatory activity is optimised by its ability to affect distinct, but equally important functions of memory and naïve T cells. Together with its role as a regulator of T cell activation and survival following T cell priming [15], CD31-mediated attenuation of memory T cell chemokinesis defines this molecule as a unique multifunctional immunoregulatory receptor.

The clinical relevance of these observations applies to a variety of inflammatory conditions including autoimmunity, allograft rejection, and the response to pathogens. CD31 polymorphisms affecting its binding site and cyoplasmic ITIMs correlate with increased GVHD and atherosclerosis severity [7], [8], [9], [10], [11], [12], [13]. It has been hypothesized that these mutations may influence CD31 binding affinity, structure and signalling capacity [36]. Hence, the possibility that these polymorphisms might affect CD31-mediated regulation of T cell function and thus contribute to the impact of T cell inflammation will require prompt investigation and careful analysis of CD31 polymorphisms in a variety of T cell mediated diseases. Importantly, a recent report that CD31 activity can be enhanced by peptide ligation in human T cells [37], together with our observations paves the way for the therapeutic manipulation of this molecule in the control of T cell-mediated inflammation.

## Methods

### Ethics Statement

This study was carried out in strict accordance with the Home Office recommendations and under its authority following approval by the Imperial College London/Central Biomedical Services ethics committee (REF. PPL 70/5872).

### Mice

CD31−/− and wild type (WT) mice were generated as previously described [2] and used at the age of 8–10 weeks.

### Reagents

APC-conjugated anti-mouse CD3 was obtained from Caltag Laboratories (Burlingame, CA, USA). Polyclonal Anti-Phospho Akt (Ser473) was obtained from Cell Signalling Technology (New England Biolabs, Hertfordshire, UK) while rat anti-mouse CD31 (clone 390), an antibody shown to interfere with CD31-mediated cell aggregation [31], was purchased from eBioscience, Ltd. (Hatfield, UK). All the other antibodies used in this study were purchased form BD Biosciences unless specified otherwise. The cell linker PKH26 was purchased from Sigma-Aldrich (Gillingham, Dorset, UK). For T cell labeling, PKH26 was added at a final concentration of 5 µM. CXCL-10, CCL19 and CCL21 chemokines were purchased from PeproTech (London, UK). Wortmannin was obtained from Sigma-Aldrich.

### HY-specific Effector Memory T Cells and Activated T Cells

Effector memory CD4^+^ and CD8^+^ T cells specific for the male-specific minor transplantation antigen HY peptide Dby epitope and restricted by H2-A^b^ and for the Uty epitope restricted by H2-D^b^, respectively, were obtained from WT and CD31−/− mice by 2 fortnightly intraperitoneal (i.p.) immunization of female mice with male splenocytes, and further expansion with male splenocytes *in vitro*, as previously described [21], [22]. Both T cell populations were composed by approximately 20% CD8^+^ and 75% CD4^+^ T cells which displayed similar phenotypes (Figure S3). These cells are referred as to effector memory T cells throughout the text.

Activated T cells were obtained by polyclonal stimulation of LN cells with plate-bound anti-CD3 (1 µg/ml, eBiosciences, Hatfield, UK) and anti-CD28 (5 µg/ml, eBiosciences, Hatfield, UK) in RPMI 1640 supplemented with 10%FCS, 2 µM glutamine, 50 IU/ml penicillin, 50 µg/ml streptomycin, 50 µM 2-mercaptoethanol, 20 µM HEPES, 1 µM sodium pyruvate and 20 U/ml recombinant IL-2 (Roche, West Sussex, UK) for 7 days at 37°C. CD31 expression by activated T cells was down regulated compared to that of naïve T cells (data not shown, and Figure 3a–d), as previously described [15], [38]. These cells are referred as to activated T cells throughout the text.

Chemokinesis assays. For chemokinesis assays, T cells were seeded (5−10×10^5^/well) in the upper chamber of a 5 µm-pore polycarbonate Transwell (Costar). A 0.7 ml volume of the chemokinesis medium (RPMI 0.5% FCS) containing either CXCL10 (300 ng/ml), or CCL19 (200 ng/ml) and CCL21 (200 ng/ml) was added to the bottom chamber, while 0.3 ml of cell suspension was added to the top chamber. Transwells were incubated for 6 hours at 37°C with 5% CO2. The number of migrated cells was evaluated using CountBright™ absolute counting beads (Molecular probes) and flow cytometry. Results are expressed as percentage of transmigrated cells.

### Peritoneal T Cell Recruitment by Chemokines

Labeled T cells (10^7^/mouse) were injected i,v, into syngeneic female mice received an i.p. injection of CXCL10 (1200 ng) immediately prior to the adoptive transfer. Enrichment of labeled T cells in the peritoneal lavage was assessed 16 hours later by flow cytometry using a FACSCalibur (Becton Dickinson, Mountain View, CA) and FlowJo version 7.1.2 software (Tree Star Inc, Ashland, OR, USA).

### Measurement of AKT/PKB Phosphorylation

Naïve or activated T cells from WT and CD31−/− mice were ‘rested’ in RPMI 1640 medium supplemented with 10% FCS at 37°C overnight before stimulation with 300 ng/ml CCL21/CCL19 for naïve T cells and 300 ng/ml CXCL-10/IP-10 for activated T cells. Cells were harvested at the indicated time points, fixed with 2% PFA for 15 min at 37°C, washed twice with PBS, permeabilized with 90% ice-cold methanol for 10 min at −20°C then washed twice with PBS. Intracellular staining was carried out after initially blocking permeabilized cells in RT FACS buffer (0.5% BSA/PBS + Na3VO4) for 30 min and incubation with a dilution of 1∶100 of Phospho-Akt (Ser473) rabbit anti-mouse antibody (Cell Signalling Technology) for 30 min at room temperature. Cells were washed and stained with secondary APC-F(ab)_2_ fragment donkey anti-rabbit IgG (H+L) (Jackson ImmunoResearch, Suffolk, UK) at 1∶100 for 30 min at room temperature. Cells were then analyzed using flow cytometry.

### Confocal Imaging

Cells were allowed to adhere onto poly-l-lysine coated coverslips, fixed in 4% paraformaldehyde and permeabilized using 0.2% Triton X-100 (Sigma-Aldrich, Gillingham, UK) in PBS. In experiments using T cells exposed to chemokines, permeabilization was not performed in order to facilitate the detection of membrane CD31. Following fixation cells were washed in PBS, blocked in blocking buffer (PBS containing 0.1%Fish skin gelatin and 1% FCS) and then stained with rabbit anti-mouse for CD31 (Novus Biologicals, Cambridge, UK) and rat anti- mouse LFA-1 (eBioscience, Ltd., Hatfield, UK). Following staining, cells were washed again and stained with secondary antibodies Alexa Fluor 488-conjugated donkey anti-rabbit IgG and Alexa Fluor 647-conjugated goat anti-rat IgG (Invitrogen, Paisley, UK). Coverslips were mounted onto slides and then examined using an Leica SP5 confocal microscope equipped with a 63×1.4 NA objective. Confocal images and z-stacks were acquired and analyzed by Leica LAS software. Repositioning of scale bars and image layouts were prepared using Adobe Photoshop. All images in a group were treated equally.

### Statistical Analysis

The Student’s t-Test test was used do assess significance of all experiments presented. All reported p-values are two-sided. A p value >0.05 was considered significant.

## Supporting Information

Figure S1 **Phenotype of WT and CD31-deficient naïve and activated T cells.** WT and CD31−/− naïve (panel a) and activated (anti-CD3 plus anti-CD28, 7 days, panel b) T cells were stained with the indicated antibodies (solid line). T cells were incubated with an isotype-matched antibody as a control (grey line). Expression of indicated molecules was analyzed by flow cytometry.
(TIF)

Figure S2 **Chemokinesis by WT and CD31−/− activated T cells in response to CXCL12.** Activated WT and CD31−/− T cell migration through a transwell in response to the chemokine CXCL12 (100 ng/ml) was assessed over 6 hours. Percentage migration was calculated by dividing the number of cells in the bottom chamber by the original number of cells plated onto the transwell. The average percentage migration from three independent experiments is shown. Error bars indicate SD (*p<0.01).
(TIF)

Figure S3 **Phenotype of HY-specific WT and CD31−/− T cells.** Expression of the molecules indicated above each set of panels by WT and CD31−/− HY-specific T cells was assessed at the time of injection (i.e., 7–10 days following re-stimulation *in vitro*) by flow cytometry. Staining with an isotype-matched control antibody is indicated by the light grey profiles.
(TIF)

## References

[1] Muller WA (2003). Leukocyte-endothelial cell interactions in leukocyte transmigration and the inflammatory response.. Trends in Immunol.

[2] Duncan GS, Andrew DP, Takimoto H, Kaufman SA, Yoshida H (1999). Genetic evidence for functional redundancy of Platelet/Endothelial cell adhesion molecule-1 (PECAM-1): CD31-deficient mice reveal PECAM-1-dependent and PECAM-1-independent functions.. Journal of immunology.

[3] Tada Y, Koarada S, Morito F, Ushiyama O, Haruta Y (2003). Acceleration of the onset of collagen-induced arthritis by a deficiency of platelet endothelial cell adhesion molecule 1.. Arthritis Rheum.

[4] Wong MX, Hayball JD, Hogarth PM, Jackson DE (2005). The inhibitory co-receptor, PECAM-1 provides a protective effect in suppression of collagen-induced arthritis.. J Clin Immunol.

[5] Groyer E, Nicoletti A, Ait-Oufella H, Khallou-Laschet J, Varthaman A (2007). Atheroprotective effect of CD31 receptor globulin through enrichment of circulating regulatory T-cells.. Journal of the American College of Cardiology.

[6] Sun J, Williams J, Yan HC, Amin KM, Albelda SM (1996). Platelet endothelial cell adhesion molecule-1 (PECAM-1) homophilic adhesion is mediated by immunoglobulin-like domains 1 and 2 and depends on the cytoplasmic domain and the level of surface expression.. The Journal of biological chemistry.

[7] Elrayess MA, Webb KE, Bellingan GJ, Whittall RA, Kabir J (2004). R643G polymorphism in PECAM-1 influences transendothelial migration of monocytes and is associated with progression of CHD and CHD events.. Atherosclerosis.

[8] Behar E, Chao NJ, Hiraki DD, Krishnaswamy S, Brown BW (1996). Polymorphism of adhesion molecule CD31 and its role in acute graft-versus-host disease.. The New England journal of medicine.

[9] Balduini CL, Frassoni F, Noris P, Klersy C, Iannone AM (2001). Donor-recipient incompatibility at CD31-codon 563 is a major risk factor for acute graft-versus-host disease after allogeneic bone marrow transplantation from a human leucocyte antigen-matched donor.. British journal of haematology.

[10] Cavanagh G, Chapman CE, Carter V, Dickinson AM, Middleton PG (2005). Donor CD31 genotype impacts on transplant complications after human leukocyte antigen-matched sibling allogeneic bone marrow transplantation.. Transplantation.

[11] El-Chennawi FA, Kamel HA, Mosaad YM, El-Sherbini SM, El-Billey NA (2006). Impact of CD31 mismatches on the outcome of hematopoeitic stem cell transplant of HLA-identical sibling.. Hematology.

[12] Goodman RS, Ewing J, Evans PC, Craig J, Poulton K (2005). Donor CD31 genotype and its association with acute graft-versus-host disease in HLA identical sibling stem cell transplantation.. Bone marrow transplantation.

[13] Auer J, Weber T, Berent R, Lassnig E, Lamm G (2003). Genetic polymorphisms in cytokine and adhesion molecule genes in coronary artery disease.. American journal of pharmacogenomics : genomics-related research in drug development and clinical practice.

[14] Newman PJ, Newman DK (2003). Signal transduction pathways mediated by PECAM-1: new roles for an old molecule in platelet and vascular cell biology.. Arterioscler Thromb Vasc Biol.

[15] Ma L, Mauro C, Cornish GH, Chai JG, Coe D (2010). Ig gene-like molecule CD31 plays a nonredundant role in the regulation of T-cell immunity and tolerance.. Proceedings of the National Academy of Sciences of the United States of America.

[16] Ross EA, Coughlan RE, Flores-Langarica A, Bobat S, Marshall JL (2011). CD31 is required on CD4+ T cells to promote T cell survival during Salmonella infection.. Journal of immunology.

[17] Graesser D, Solowiej A, Bruckner M, Osterwei E, Juedes A (2002). Altered vascular permeability and early onset of experimental autoimmune encephalomyelitis in PECAM-1-deficient mice.. J Clin Invest.

[18] Ward SG, Marelli-Berg FM (2009). Mechanisms of chemokine and antigen-dependent T-lymphocyte navigation.. Biochem J.

[19] Donath C, Grassel E, Baier D, Pfeiffer C, Karagulle D (2011). Alcohol consumption and binge drinking in adolescents: comparison of different migration backgrounds and rural vs. urban residence–a representative study.. BMC public health.

[20] David R, Ma L, Ivetic A, Takesono A, Ridley AJ (2009). T-cell receptor- and CD28-induced Vav1 activity is required for the accumulation of primed T cells into antigenic tissue.. Blood.

[21] Marelli-Berg FM, James MJ, Dangerfield J, Dyson J, Millrain M (2004). Cognate recognition of the endothelium induces HY-specific CD8+ T-lymphocyte transendothelial migration (diapedesis) in vivo.. Blood.

[22] Jarmin SJ, David R, Ma L, Chai J-G, Dewchand H (2008). Targeting T cell receptor-induced phosphoinositide-3-kinase p110delta activity prevents T cell localization to antigenic tissue.. J Clin Invest.

[23] Bucy RP, Panoskaltsis-Mortari A, Huang GQ, Li J, Karr L (1994). Heterogeneity of single cell cytokine gene expression in clonal T cell populations.. The Journal of experimental medicine.

[24] Wells AD, Gudmundsdottir H, Turka LA (1997). Following the fate of individual T cells throughout activation and clonal expansion. Signals from T cell receptor and CD28 differentially regulate the induction and duration of a proliferative response.. The Journal of clinical investigation.

[25] Gudmundsdottir H, Wells AD, Turka LA (1999). Dynamics and requirements of T cell clonal expansion in vivo at the single-cell level: effector function is linked to proliferative capacity.. Journal of immunology.

[26] Nombela-Arrieta C, Lacalle RA, Montoya MC, Kunisaki Y, Megias D (2004). Differential requirements for DOCK2 and phosphoinositide-3-kinase gamma during T and B lymphocyte homing.. Immunity.

[27] Evans R, Patzak I, Svensson L, De Filippo K, Jones K (2009). Integrins in immunity.. Journal of cell science.

[28] Nieto M, Frade JM, Sancho D, Mellado M, Martinez AC (1997). Polarization of chemokine receptors to the leading edge during lymphocyte chemotaxis.. The Journal of experimental medicine.

[29] Wu Y, Stabach P, Michaud M, Madri JA (2005). Neutrophils lacking platelet-endothelial cell adhesion molecule-1 exhibit loss of directionality and motility in CXCR2-mediated chemotaxis.. Journal of immunology.

[30] Zhao T, Newman PJ (2001). Integrin activation by regulated dimerization and oligomerization of platelet endothelial cell adhesion molecule (PECAM)-1 from within the cell.. The Journal of cell biology.

[31] Baldwin HS, Shen HM, Yan HC, DeLisser HM, Chung A (1994). Platelet endothelial cell adhesion molecule-1 (PECAM-1/CD31): alternatively spliced, functionally distinct isoforms expressed during mammalian cardiovascular development.. Development.

[32] Manes TD, Hoer S, Muller WA, Lehner PJ, Pober JS (2010). Kaposi’s sarcoma-associated herpesvirus K3 and K5 proteins block distinct steps in transendothelial migration of effector memory CD4+ T cells by targeting different endothelial proteins.. Journal of immunology.

[33] Pumphrey NJ, Taylor V, Freeman S, Douglas MR, Bradfield PF (1999). Differential association of cytoplasmic signalling molecules SHP-1, SHP-2, SHIP and phospholipase C-gamma1 with PECAM-1/CD31.. FEBS Lett.

[34] Harris SJ, Parry RV, Foster JG, Blunt MD, Wang A (2011). Evidence that the lipid phosphatase SHIP-1 regulates T lymphocyte morphology and motility.. Journal of immunology.

[35] Marelli-Berg FM, Cannella L, Dazzi F, Mirenda V (2008). The highway code of T cell trafficking.. J Pathol.

[36] Goodman RS, Kirton CM, Oostingh GJ, Schon MP, Clark MR (2008). PECAM-1 polymorphism affects monocyte adhesion to endothelial cells.. Transplantation.

[37] Fornasa G, Groyer E, Clement M, Dimitrov J, Compain C (2010). TCR Stimulation Drives Cleavage and Shedding of the ITIM Receptor CD31.. J Immunol.

[38] Bird IN, Spragg JH, Ager A, Matthews N (1993). Studies of lymphocyte transendothelial migration: analysis of migrated cell phenotypes with regard to CD31 (PECAM-1), CD45RA and CD45RO.. Immunology.

