# Supplementary figures and images for: Primed T Cell Responses to Chemokines Are Regulated by the Immunoglobulin-Like Molecule CD31
Source: PLoS One. 2012 Jun 19;7(6):e39433. doi: 10.1371/journal.pone.0039433 (PMC3378580; doi:10.1371/journal.pone.0039433)

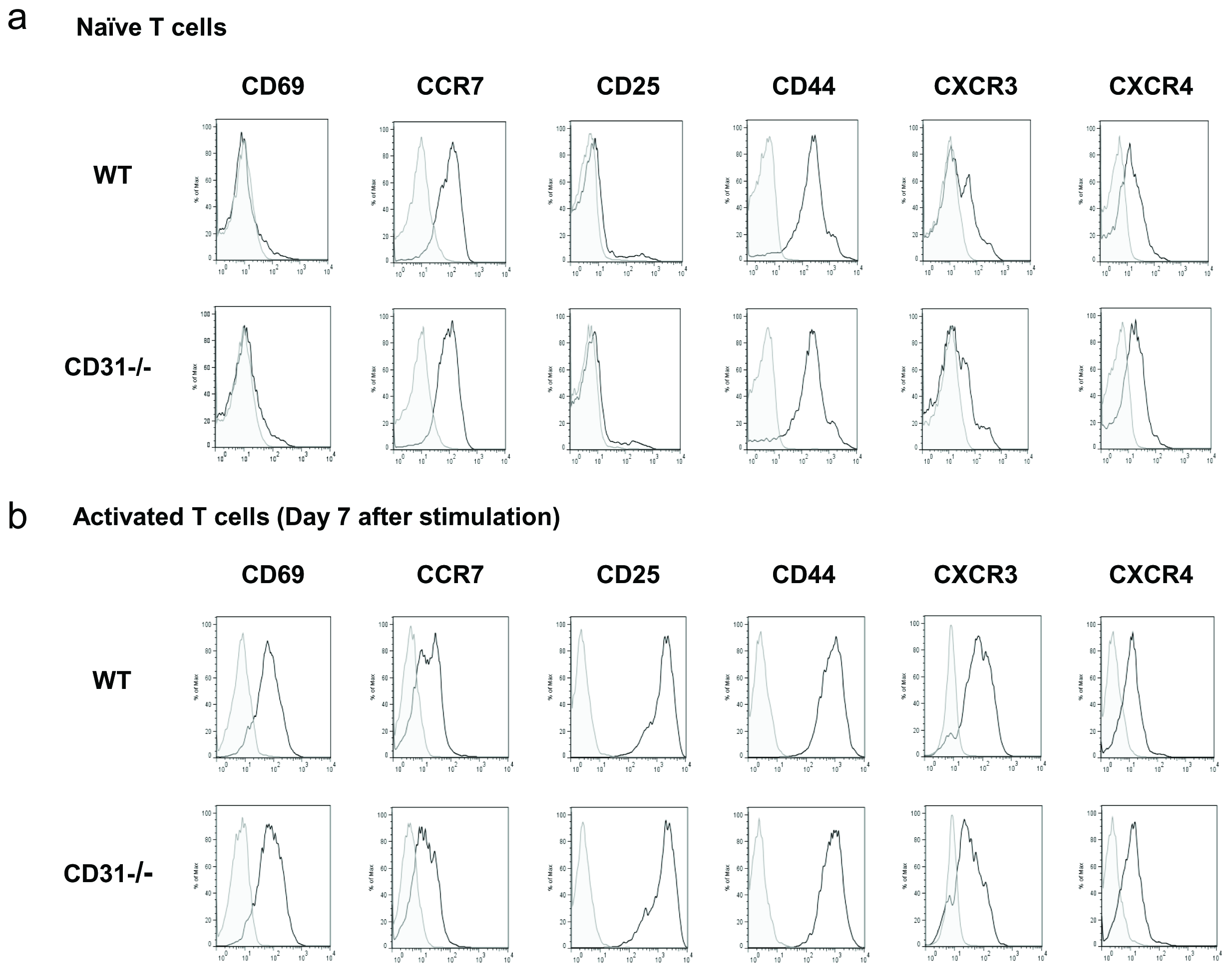

Supplement: Figure S1 — Phenotype of WT and CD31-deficient naïve and activated T cells. WT and CD31−/− naïve (panel a) and activated (anti-CD3 plus anti-CD28, 7 days, panel b) T cells were stained with the indicated antibodies (solid line). T cells were incubated with an isotype-matched antibody as a control (grey line). Expression of indicated molecules was analyzed by flow cytometry. (TIF) [file pone.0039433.s001.tif]

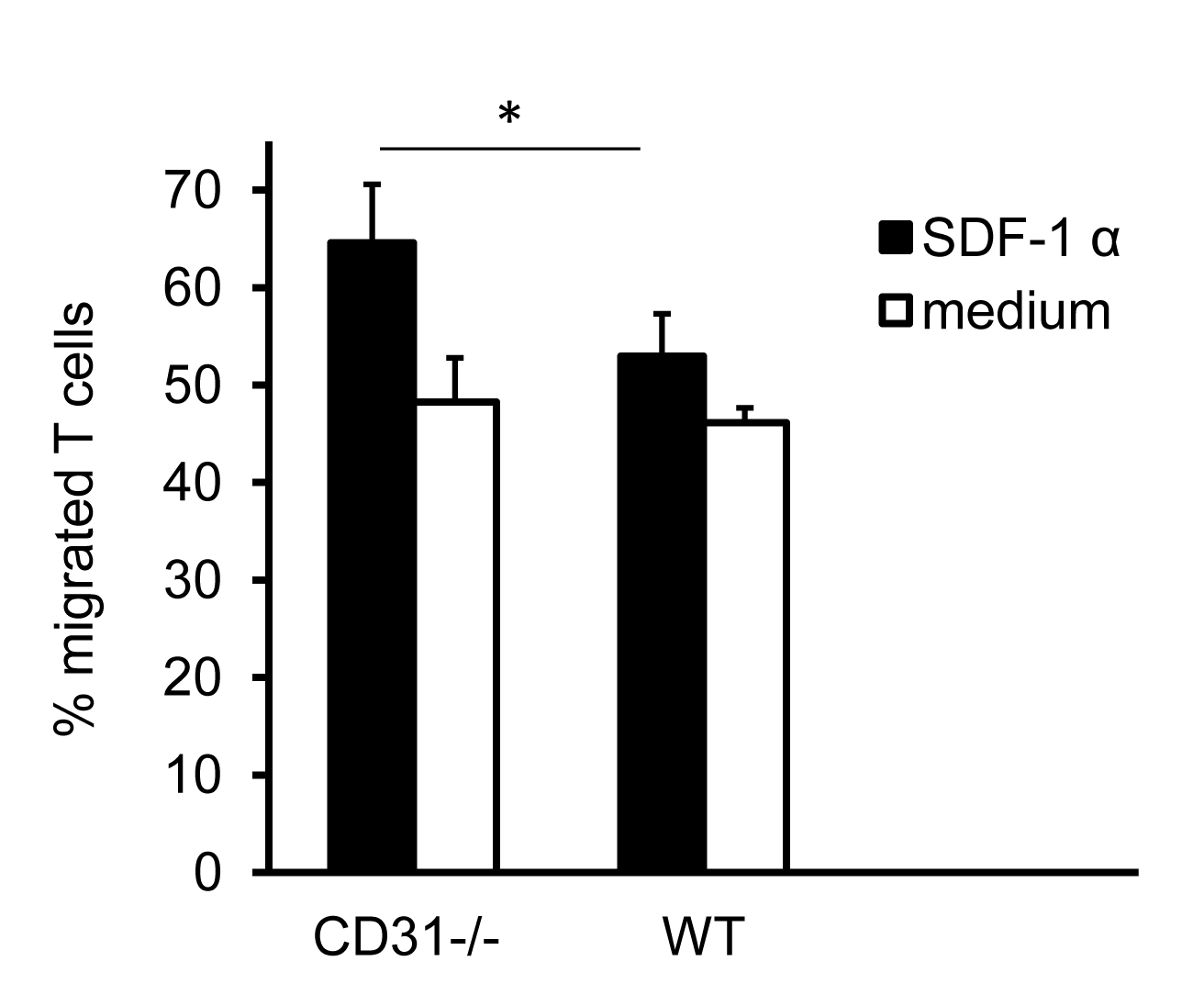

Supplement: Figure S2 — Chemokinesis by WT and CD31−/− activated T cells in response to CXCL12. Activated WT and CD31−/− T cell migration through a transwell in response to the chemokine CXCL12 (100 ng/ml) was assessed over 6 hours. Percentage migration was calculated by dividing the number of cells in the bottom chamber by the original number of cells plated onto the transwell. The average percentage migration from three independent experiments is shown. Error bars indicate SD (*p<0.01). (TIF) [file pone.0039433.s002.tif]

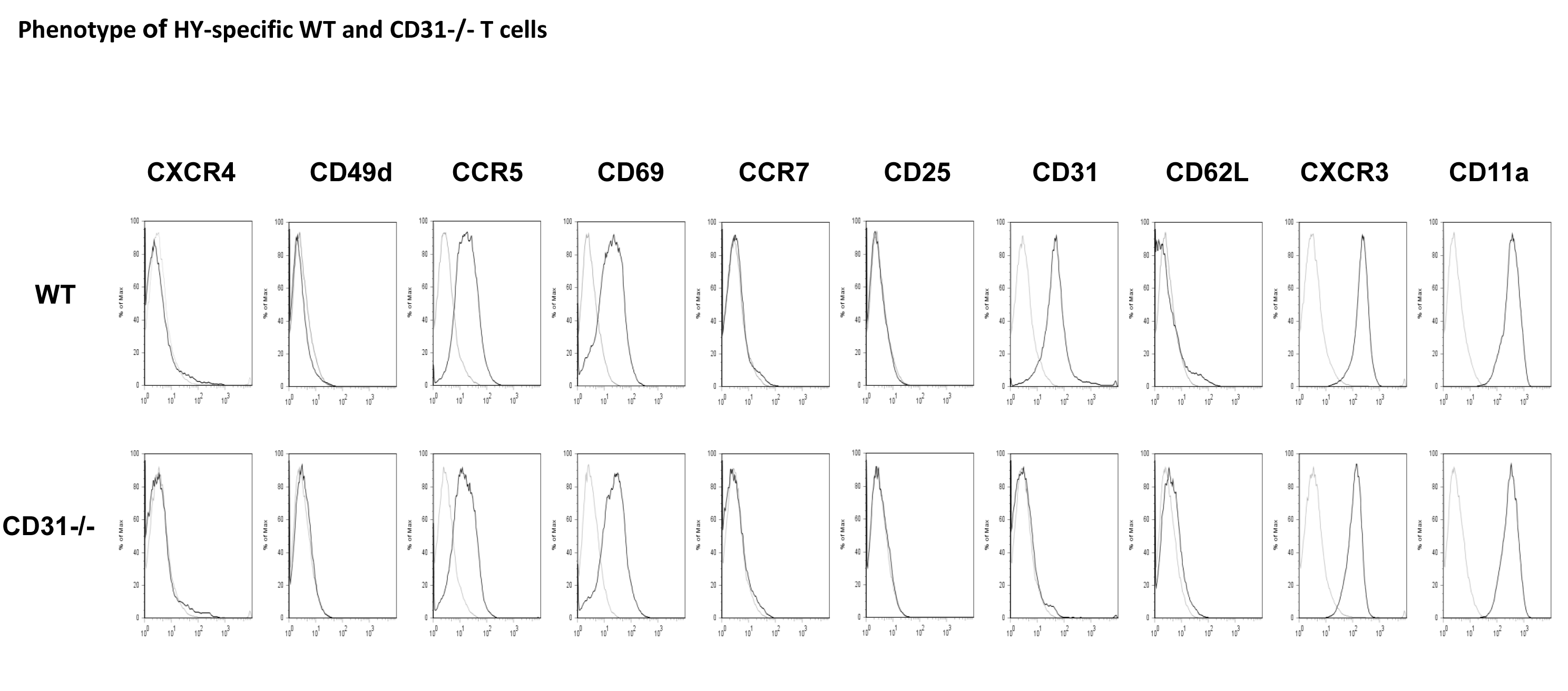

Supplement: Figure S3 — Phenotype of HY-specific WT and CD31−/− T cells. Expression of the molecules indicated above each set of panels by WT and CD31−/− HY-specific T cells was assessed at the time of injection (i.e., 7–10 days following re-stimulation in vitro) by flow cytometry. Staining with an isotype-matched control antibody is indicated by the light grey profiles. (TIF) [file pone.0039433.s003.tif]
